# Comparing the effects of retro and forward walking on serum adiponectin levels in obese young adults

**DOI:** 10.1016/j.jtumed.2023.01.009

**Published:** 2023-01-28

**Authors:** Khalid Alkhathami, Ajith Soman, Sunil Chandy, Baranitharan Ramamoorthy, Bijad Alqahtani

**Affiliations:** a Department of Health Rehabilitation, College of Applied Medical Sciences, Shaqra University, Shaqra, KSA; b Department of Clinical Lab Science, College of Applied Medical Sciences, Shaqra University, Shaqra, KSA

**Keywords:** التدريب على المشي, المشي الرجعي, الأديبونكتين, علامات الالتهابات, مؤشرات السمنة, Adiponectin, Inflammatory markers, Obesity indices, Retro walking, Treadmill training

## Abstract

**Objectives:**

Retro walking or backward walking expends greater energy and places less stress on joints compared with forward walking at a similar speed. This study conducted in obese young men was primarily aimed at comparing the effects of backward walking with forward walking on adiponectin levels. The secondary aim was to describe the effects of concomitant factors, namely C-reactive protein, body mass index (BMI), waist to height ratio, and waist to hip ratio, on adiponectin levels in obese young men.

**Methods:**

In this randomized comparative study, 102 participants underwent either retro walking or forward walking treadmill training four times a week for 12 weeks before and after which adiponectin, C-reactive protein, BMI, waist to height ratio, and waist to hip ratio were measured. Comparison of the measured values before and after intervention and between the groups was done, and the influence of C-reactive protein, BMI, waist to height ratio, and waist to hip ratio on adiponectin levels was determined.

**Results:**

Adiponectin levels were significantly increased (p < 0.001) and C-reactive protein, BMI, waist to height ratio, and waist to hip ratio were significantly decreased (p ≤ 0.001) post-intervention. The participants who underwent retro walking training showed a significantly higher change in C-reactive protein levels, BMI, and waist to hip ratio compared to the forward walking group (p < 0.001). Adiponectin levels were influenced by BMI (p < 0.001).

**Conclusion:**

Retro walking training leads to a greater increase in adiponectin and reduction in C-reactive protein, BMI, waist to height ratio, and waist to hip ratio compared to forward walking, and adiponectin levels are influenced by BMI. Retro walking treadmill training can be preferentially used to decrease cardiovascular risk factors.

## الملخص

**أهداف البحث:**

يستهلك المشي الرجعي أو المشي للخلف طاقة أكبر ويضع ضغطا أقل على المفاصل مقارنة بالمشي إلى الأمام بسرعة مماثلة. هدفت هذه الدراسة التي أجريت على الشباب البدينين في المقام الأول إلى مقارنة تأثيرات المشي إلى الوراء مع تأثيرات المشي إلى الأمام على مستويات الأديبونكتين. كان الهدف الثانوي هو وصف تأثيرات العوامل المصاحبة وهي البروتين المتفاعل-سي، ومؤشر كتلة الجسم، ونسبة الخصر إلى الطول ونسبة الخصر إلى الورك على مستويات الأديبونيكتين لدى الشباب البدينين.

**طريقة البحث:**

في هذه الدراسة التجريبية العشوائية المقارنة، خضع مائة واثنان من المشاركين إما للتدريب على المشي الرجعي أو المشي للأمام أربع مرات في الأسبوع لمدة اثني عشر أسبوعا قبل وبعد ذلك أديبونيكتين، البروتين المتفاعل-سي، مؤشر كتلة الجسم، نسبة الخصر إلى الطول، ونسبة الخصر إلى الورك تم قياسها. تم إجراء مقارنة بين القيم المقاسة قبل وبعد التدخل وبين المجموعات وتم تحديد تأثير البروتين المتفاعل-سي ومؤشر كتلة الجسم ونسبة الخصر إلى الطول ونسبة الخصر إلى الورك على مستويات الأديبونكتين.

**النتائج:**

زادت مستويات أديبونيكتين بشكل ملحوظ وانخفض البروتين المتفاعل-سي ومؤشر كتلة الجسم ونسبة الخصر إلى الطول ونسبة الخصر إلى الورك بشكل ملحوظ بعد التدخل. أظهر المشاركون الذين خضعوا لتدريب المشي الرجعي تغيرا أعلى بشكل ملحوظ في مستويات البروتين المتفاعل-سي، ومؤشر كتلة الجسم ونسبة الخصر إلى الورك مقارنة بمجموعة المشي إلى الأمام. شوهدت مستويات أديبونيكتين تتأثر بمؤشر كتلة الجسم

**الاستنتاجات:**

يؤدي تدريب المشي الرجعي إلى زيادة أكبر في الأديبونيكتين وتقليل البروتين المتفاعل-سي، ومؤشر كتلة الجسم، ونسبة الخصر إلى الطول ونسبة الخصر إلى الورك مقارنة بالمشي إلى الأمام، وتتأثر مستويات الأديبونكتين بمؤشر كتلة الجسم. يمكن استخدام التدريب على جهاز المشي الرجعي بشكل تفضيلي لتقليل عوامل الخطر القلبية الوعائي.

## Introduction

Obesity is a problem faced worldwide, which appears to affect young adults more than any other age group.^1^ The fact that much of this weight gain occurs in those already obese or overweight is a source of concern. The term young adults is defined in the literature in various ways, commonly with a lower limit of 15 years, but with varying upper limits of 24, 29, and 39 years of age.^1^, ^2^, ^3^, ^4^

The risk of weight gain in college-aged young adults ranging from 19 to 29 years is considerable and has been attributed to major lifestyle changes that occur around this age such as moving away from home, increased consumption of restaurant food, decreased physical activity, increased sedentary behavior, and being prone to binging on unhealthy foods.^5^, ^6^, ^7^ This increase in body weight can negatively affect quality of life due to the perception that fit and thin bodies are attractive, and also pose a public health challenge due to the poor health impacts of overweight and obesity.^8^^,^^9^

Adiponectin belonging to the family of adipokines and produced predominantly by adipocytes, has recently attracted the attention of the scientific community due to its anti-inflammatory, cardioprotective, anti-atherogenic, and antidiabetic effects.^10^ Previously thought to be produced exclusively by adipose tissue, adiponectin was later proven to also be produced by other tissues including liver parenchyma cells, epithelial cells, placental tissue, and human and murine osteoblasts. A fall in blood adiponectin levels correlates with cardiovascular complications of obesity such as peripheral artery disease and ischemic heart disease and many cancer types.^11^, ^12^, ^13^, ^14^ Adiponectin concentrations are reduced in metabolic syndrome and obesity, and possibly have a role in the development of insulin resistance. In addition, weight loss causes an increase in adiponectin concentrations. Adiponectin has the potential to act as a protective endocrine/autocrine/paracrine factor to prevent and halt the progression of potentially life-threatening complications of obesity.^15^, ^16^, ^17^ Levels of obesity and adiponectin have an inverse correlation, and adiponectin levels are tightly regulated at the transcriptional and translational levels.^18^

Exercise has the potential to alter adiponectin levels. Studies have shown that adiponectin concentrations decrease after high-intensity aerobics but remain unchanged after moderate-intensity aerobic exercise in middle-aged persons with abdominal obesity.^19^ Another recent study showed that 10 weeks of interval training in the aerobic mode could increase the plasma level of adiponectin in young men, whereas these levels decreased with 4 weeks of detraining.^20^ Circulating adiponectin level can be influenced by body composition changes unrelated to exercise, which means that weight reduction solely based on a diet with reduced calories can also decrease insulin resistance and increase total adiponectin levels.^21^

Exercise and physical activity are essential in controlling obesity, and brisk walking is one of the most recommended exercises for reducing fat and improving cardiovascular health.^22^ Backward walking or retro walking on a treadmill expends higher energy than forward walking. This is postulated to be due to shorter strides taken at increased frequencies, which increases the metabolic cost.^23^ Backward walking consumes more energy than forward walking at similar speeds, and oxygen consumption and heart rate increase further with the speed at which backward walking is done. There is scientific proof documenting that retro walking causes more energy expenditure than normal walking.^24^, ^25^, ^26^

It is a well-documented fact that backward walking causes more energy expenditure with less biomechanical stress on the joints, but it has not been studied whether backward walking has an effect on modifying adiponectin levels. There is a dearth of evidence on how retro walking training affects adiponectin levels. Thus, the present study assessed the effects of a 12-week retro walking treadmill training program compared to those of a forward walking program on serum adiponectin levels in obese young men. We hypothesized that adiponectin levels change differently between subjects who undergo retro walking and forward walking.

## Materials and Methods

### Study design

The study was a randomized comparative study in young men enrolled in medical and paramedical courses at Shaqra University, KSA. The participants were solicited by advertisements placed on the university notice boards and volunteered to participate in the study. Clearance was obtained from the University Clinical Committee, and the principles outlined in declaration of Helsinki were met when conducting the study.

### Participants and recruitment

Men between 18 and 25 years of age were included in the study, who had a body mass index (BMI) ≥ 30 kg/m^2^. Athletes; people with diabetes; smokers; those on glucose-lowering medication; those who had any orthopedic, respiratory, neurological, or metabolic conditions contraindicating exercise training; and those currently on drug therapy were excluded. Based on the parameters used in a previous study and using a 95% confidence interval and 90% power, a sample size of 29 in each group was estimated.^27^ Allowing for a dropout rate of 10% and considering the number of participants who volunteered for participation, 131 men were screened for eligibility of whom 7 were excluded as they declined to give consent. After initial screening, randomization was done for 114 participants and 57 were included in each group. After discounting dropouts, final analyses were done for 49 in the retro walking group and 53 in the forward walking group.

Initial data collected from the participants included age, sex, education, and clinical parameters such as weight, height, BMI, hip circumference (HC), waist circumference (WC), waist to height ratio (WHtR), and waist to hip ratio (WHR), which were measured or calculated. Physical activity profile and any history of past illness were noted and a full physical examination was conducted. Anyone who did not fulfill the inclusion criteria was removed from the study.

Prior to initial assessment, the participants were given a data package with the details of the study, which contained their rights as research project participants and a printed informed consent form. The procedure was thoroughly explained to the participants and any doubts were clarified before they signed the consent form. After signing the forms, the participants were designated to one of two groups randomly using sequentially numbered and sealed opaque envelopes, and the respective assessments and interventions were conducted. The participants' diet remained the same throughout the duration of the study. Screening was done using the preparticipation questionnaire published by the American College of Sports Medicine/American Heart Association.^28^

### Calculation of clinical data

The weight and height of the participants were measured using a portable electronic weighing machine and portable stadiometer, respectively. When measuring height, the feet were placed together, the head was positioned in the Frankfurt horizontal plane and shoulder blades, and the buttocks and heels were placed against the stick. The Quetelet Index was used to calculate BMI in kg/m^2^.^29^ The World Health Organization recommended method was used for measuring WC, HC, and height and for calculating WHR and WHtR. WC measurement was done midway between the lower margin of the ribcage and upper margin of the iliac crest at the end of a normal expiration.^30^^,^^31^ HC measurement was taken from the widest part of the hip or buttocks at the level of greater trochanter. Two measurements each of the WC and HC were taken with as less clothing as possible, standing relaxed and erect, arms at the side and feet together. Anthropometric tape was used for measuring to the nearest 0.1 cm. WHR, the ratio of waist circumference to hip circumference, was calculated by dividing the WC by the HC measured in same units. WHtR was obtained by dividing WC by length.^32^^,^^33^

### Exercise protocol

Prior to starting the intervention, the participants who were unfamiliar with the exercise received basic training in forward and backward walking treadmill walking, until they were confident in walking both ways on the treadmill without support. At the onset, the formula 208-(0.7 × age) was used to calculate the maximal heart rate.^34^ Four minutes each of warm up and cool down exercises were given before and after the intervention for all participants. These exercises included free exercises of all joints, hamstring and soleus stretching, and heel raise exercises. Random allocation to either forward walking or retro walking groups was done using a sequentially numbered envelope.

The exercise training given to the participants in the retro walking group was a supervised backward walking treadmill program done at a frequency of 4 days a week for a duration of 12 weeks. Apart from the warm up and cool down times, each session had an exercise time that was initially 15 min and progressed to 30 min over a period of 6 weeks. The exercise period consisted of backward walking at a pace of 4 km/h (67 m/min at a gradient of 10%).^35^^,^^36^ The subjects in the forward walking group went through a forward walking treadmill training program, also under supervision, with intensity, duration, and frequency similar to the retro walking exercise training program. The outcome measurements were done before commencement and after the end of the exercise training program.

### Blood sample collection and analyses

Blood samples were collected after a 12-h fast, done overnight. Then 10 mL blood was collected from the antecubital vein immediately before the first and 24–72 h after the last treadmill training session. An enzyme-linked immunosorbent assay (B-Bridge International, Inc., San Jose, CA) of whole plasma stored at −87 °C until use was used to determine adiponectin levels in the plasma.^37^ C-reactive protein (CRP) levels were determined using the fluorescence immunochromatographic method (Wiz biotech®).^38^ The assessors who measured the outcomes received training from the research team and were blinded to the phase of measurement (preintervention or postintervention), to the aims and objectives of the study, and to the training groups to which the subjects belonged.

### Statistical analyses

The collected data were summarized by descriptive statistics, i.e., percentage, frequency, mean, and standard deviation. When tested for normality using the Shapiro–Wilks method, the data were found to be normally distributed, and hence parametric methods were used for the analyses. The paired *t*-test was used to compare the outcomes before intervention and after. The independent *t*-test was used to examine the differences in the demographic data between the two groups. Pearson's correlation coefficient was used to find the relationship between adiponectin with other variables. Multiple linear regression analyses were performed to identify the influence of independent variables (BMI, WHtR, WHR, and CRP) on the dependent variable (adiponectin). All analyses were carried out using SPSS version 20.0 (IBM Co., Armonk, NY, USA) with the level of significance set at p < 0.05.

## Results

**Figure 1:**
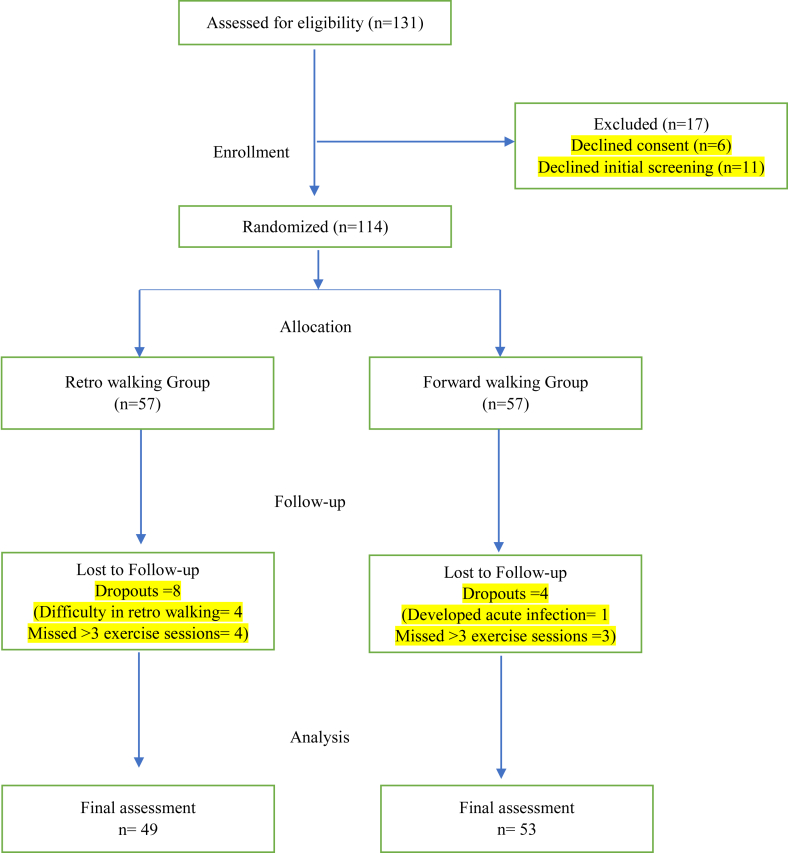
CONSORT flowchart.

In total, 131 participants were assessed for their eligibility to participate in the study, of whom, 17 were excluded for not satisfying the inclusion criteria. One hundred and fourteen subjects were randomized into one of two groups, of whom twelve dropped out during the course of the intervention (difficulty retro walking = 7, missed more than 3 consecutive exercise sessions = 5). Final data were recorded from 102 subjects: 53 in the forward walking group and 49 in the retro walking group. Figure 1 presents a CONSORT flow chart. Within group comparisons of age, BMI, WHR, WHtR, CRP, and adiponectin were done using the paired *t*-test. A significant difference (p < 0.05) was seen in the mean baseline WHtR between the groups (Table 1). There was no difference (p > 0.05) in pretest mean age, BMI, WHR, CRP, and adiponectin between the groups.

**Table 1:** Baseline outcomes of the groups.

Variable	Retro walking (n = 49)	Forward walking (n = 53)	*t*	P value
Age	21.39 ± 1.54	21.28 ± 1.69	0.326	0.745
BMI	33.92 ± 2.80	33.72 ± 3.76	0.305	0.761
WHR	0.89 ± 0.12	0.86 ± 0.07	1.488	0.140
WHtR	0.55 ± 0.08	0.59 ± 0.07	−2.509	0.014∗
CRP	3.73 ± 2.54	3.74 ± 2.24	0.030	0.977
Adiponectin	16.02 ± 2.68	14.96 ± 3	1.893	0.061

∗Significant p<0.05.

Abbreviations: BMI: Body Mass Index, WHR: Waist to hip ratio, WHtR: Waist to height ratio, CRP: C-reactive protein.

The paired *t*-test was used for the within group comparisons of BMI, WHR, WHtR, CRP, and adiponectin (Table 2). There was a difference (p < 0.05) in the mean BMI, WHtR, WHR, CRP, and adiponectin within both groups.

**Table 2:** Within group analyses of outcomes in retro walking and forward walking.

Variable		Retro walking (n = 49)				Forward walking (n = 53)			
		Mean	SD	*t*	p value	Mean	SD	*t*	P value
BMI	Pre	33.92	2.80	20.244	<0.001∗	33.72	3.76	16.724	<0.001∗
	Post	27.38	3.57			30.15	4.55		
WHR	Pre	0.89	0.12	8.385	<0.001∗	0.86	0.07	13.095	<0.001∗
	Post	0.78	0.09			0.83	0.06		
WHtR	Pre	0.55	0.08	9.893	<0.001∗	0.59	0.07	11.96	<0.001∗
	Post	0.49	0.06			0.54	0.06		
CRP	Pre	3.73	2.54	7.910	<0.001∗	3.74	2.24	6.153	<0.001∗
	Post	1.56	1.09			3.27	2.13		
Adiponectin	Pre	16.02	2.68	−17.767	<0.001∗	14.96	3.00	−21.254	<0.001∗
	Post	16.73	2.74			15.51	2.98		

∗Significant p<0.05.

Abbreviations: BMI: Body mass index, WHR: Waist to hip ratio, WHtR: Waist to height ratio, CRP: C-reactive protein.

The mean difference in outcomes between the groups were compared using the independent samples *t*-test (Table 3). The mean difference in BMI, WHR, CRP, and adiponectin significantly differed (p < 0.05) between the groups.

**Table 3:** Between group analyses (pre–post) of outcomes.

Variable	Pre–post (n = 102)	Mean	SD	*t*	P value
BMI	Retro walking	6.54	2.26	7.771	<0.001∗
	Forward walking	3.57	1.55		
WHR	Retro walking	0.10	0.09	6.322	<0.001∗
	Forward walking	0.03	0.02		
WHtR	Retro walking	0.05	0.04	0.656	0.513
	Forward walking	0.05	0.03		
CRP	Retro walking	2.17	1.92	6.153	<0.001∗
	Forward walking	0.47	0.56		
Adiponectin	Retro walking	−0.71	0.28	−3.224	0.002∗
	Forward walking	−0.56	0.19		

∗Significant p<0.05.

Abbreviations: BMI: Body mass index, WHR: Waist to hip ratio, WHtR: Waist to height ratio, CRP: C-reactive protein.

The relationship between adiponectin and the preintervention values of age, BMI, WHtR, WHR, and CRP was tested using Pearson's correlation coefficient. There was a negative linear relationship (p < 0.05) between adiponectin levels and BMI, WHtR, WHR, and CRP (Table 4).

**Table 4:** Relationship between adiponectin and other variables.

Variable	Pearson's correlation	P value
Age	0.863	0.102
BMI	−0.833	<0.001∗
WHR	−0.479	<0.001∗
WHtR	−0.562	<0.001∗
CRP	−0.414	<0.001∗

∗Significant p<0.05.

Abbreviations: BMI: Body mass index, WHR: Waist to hip ratio, WHtR: Waist to height ratio, CRP: C-reactive protein.

Multiple linear regression analysis was used to predict the influence of independent variables on adiponectin. The independent variables were preintervention values of BMI, WHtR, WHR, and CRP. For every unit increment in BMI, the adiponectin value decreased by 0.77 (p < 0.05) (Table 5).

**Table 5:** Multiple linear regression analysis of factors influencing adiponectin.

	Unstandardized coefficients		Standardized coefficients	*t*	P value
	B	Std. Error	Beta		
BMI	−0.77	0.07	−0.89	−10.608	<0.001∗
WHR	0.28	2.18	0.01	0.131	0.896
WHtR	−1.12	2.92	−0.030	−0.385	0.701
CRP	0.15	0.09	0.12	1.705	0.091
Constant	41.49	2.00	–	20.716	<0.001∗

∗Significant p<0.05.

Abbreviations: BMI: Body mass index, WHR: Waist to hip ratio, WHtR: Waist to height ratio, CRP: C-reactive protein.

## Discussion

Considering the increase in lifestyle diseases among the youth, studies that endeavor to provide evidence about non-pharmacological interventions to prevent and control these diseases have immense value, and it was with this focus that the present study was designed.

The primary aim of our study was to compare the effect that retro walking and forward walking had on plasma adiponectin levels in obese young men. The results demonstrated that adiponectin levels increased favorably in both groups, but more so in the group that underwent retro walking training. In addition, BMI, CRP, WHtR, and WHR were analyzed, and they were reduced in both normal walking and retro walking training groups, and again more so in the retro walking group in the case of BMI, WHR, and CRP. The variables BMI, WHR, WHtR, and CRP had a negative linear relationship with adiponectin levels, and high BMI or body fat was able to predict low levels of adiponectin.

Adipokines are pro-inflammatory factors, which are secreted by dysfunctional adipose tissue and have detrimental effects on the cardiovascular system. Of these adipokines, adiponectin has several beneficial effects on cardiovascular health and insulin sensitivity.^39^ It is a novel adipose-specific collagen that plays an important role in metabolic disorders. In this study, adiponectin level was seen to significantly increase in participants trained in either mode of walking, and to a greater extent in those who underwent retro walking training. Previous studies have also reported that an acute increase in adiponectin level can be caused by vigorous exercise. That said, there is a lack of consensus on the effects of exercise on adiponectin in obese adults. A review by Bouassida et al.^40^ reported that short- and long-term exercise has disparate effects on adiponectin level responses in sedentary and trained subjects. A comparison of adiponectin levels in adolescent boys with obesity who underwent aerobic or resistance exercise training, it was seen that adiponectin levels increased only in those who underwent aerobic exercise.^41^

In this research, the primary focus was to determine if a 12-week training program of retro and forward walking had effects on adiponectin levels, and if the effect differed between the interventions. We postulated that the type of exercise that caused a greater increase in adiponectin levels would be the one that better alleviates obesity-induced hypertension and endothelial dysfunction and prevents complications such as myocardial infarction, diabetic cardiomyopathy, and atherosclerosis. Some studies have found that adiponectin increases even up to 260% with exercise of sufficient duration and intensity.^42^^,^^43^ Adiponectin controls metabolic dysfunction, and interventions that restore it to normal levels can contribute to better health status by improving oxidation of fatty acids, insulin sensitivity, and glucose homeostasis.

Retro walking, in addition to being a novel way of exercising and thus able to interest people, especially young people, places a higher demand on sensorimotor, cardiovascular, metabolic, and perceptual responses than forward walking.^35^ Exercise prescription for obese individuals is a domain that most clinicians tread on carefully, because of the risk of musculoskeletal injuries to which obese persons are prone. There is always a need to identify newer and safer, yet effective ways for obese persons to exercise. Retro walking decreases eccentric activity of the quadriceps muscle at the same time maintaining isometric and concentric activity. This reduced eccentric activity decreases compressive forces on the knee joint, thus making it less prone to injury.

In our study, 12 weeks of training in young adults who were obese favorably improved plasma adiponectin levels and body fat indicators, with markedly better improvement seen in those who underwent retro walking training than in those who underwent forward walking training. The type and intensity of physical exercise seem to directly influence serum adiponectin concentrations. Obese and overweight individuals have lower adiponectin levels, which makes them prone to cardiovascular and metabolic diseases.^44^^,^^45^ An exercise such as retro walking, which can increase adiponectin levels, can be recommended for modifying the risk of morbidity and mortality in those who are obese and overweight.

The exact relationship between adiponectin levels and exercise is not fully clear, and no general conclusions have been drawn. Yet it is clear that there are many factors that influence changes in adiponectin such as duration, types and intensity of exercise, body mass, health status, age, and sex of the participants. Unlike the other adipokines and although produced by adipose tissue, adiponectin levels are paradoxically lower in persons with than without obesity. This suggests that adipose tissue exerts a negative influence on adiponectin production.^46^ This has been corroborated by our results showing a negative linear relationship between plasma adiponectin and BMI, WHtR, WHR, and CRP. Changes in body weight or body composition can cause alterations in adiponectin levels, irrespective of whether these changes have been brought about by dietary modifications, exercise training, or a combination of both.^47^^,^^48^ Serum levels of inflammatory markers are significantly higher in obese than lean subjects,^49^ which would explain the correlation between decreased adiponectin levels and increased CRP levels, both of which are important inflammatory markers, observed in the present study.

We found retro walking to be better than forward walking in improving all the obesity and inflammatory markers measured in the participants. These results add to the existing body of literature by providing evidence on the benefits of backward walking on increasing adiponectin, which has antidiabetic, antiatherogenic, cardioprotective, and anti-inflammatory effects.^10^ Adding to the equation the benefits that retro walking have in preventing injuries to the joints of the lower limb, it can be surmised that retro walking is a feasible alternative to forward walking when the aims of training are to increase levels of adiponectin and decrease body fat, thus improving cardiovascular and metabolic health.

Backward walking involves a simple temporal reversal of the kinetic and kinematic activation pattern to that seen in forward walking. The joint angle patterns for backward walking are reversed, and the moment patterns are similar to those observed in forward walking, when the speed is the same.^50^ For most of the participants, walking backwards was a novel task, and during the initial training, many of the participants conveyed apprehension in walking backwards, especially on the treadmill.

The strengths of our study were the relatively large sample size and the documentation of how retro walking influences adiponectin levels, which is a lesser used outcome measure in studying the effect of retro walking on obese young adults. The limitations included a lack of a control group, which would serve to measure any fluctuations in adiponectin when not undergoing any exercise program, non-use of any objective measure such as oxygen consumption for quantifying intensity of exercise, and lack of documentation of diet of the participants, which may have affected adiponectin levels. In addition, not including women as study participants may have affected the generalizability of the study findings across sexes.

## Conclusion

Both retro walking and forward walking can help to favorably modify adiponectin level and obesity and a retro walking program has added benefits over a forward walking program of similar intensity in modifying these outcomes. BMI, WHR, WHtR, and CRP exhibit a negative linear relationship with adiponectin. BMI was a significant predictor for adiponectin. These factors can be of value during the design of an exercise and physical activity program for modifying cardiovascular risk in young obese adults. Due to the advantages and practicability of application, retro walking can be a valuable added to any exercise program aiming to modify obesity and cardiovascular risk. Additional studies could assess the effects of retro walking on more inflammatory markers and whether these effects can be reproduced in both sexes and different age groups.

## Source of funding

The authors extend their appreciation to the Deputyship for Research and Innovation, Ministry of Education in KSA for funding this research work through project number IFP2021-071.

## Conflict of interest

The authors have no conflicts of interest to declare.

## Ethical approval

The study was conducted according to the guidelines of the Declaration of Helsinki. Approval to conduct the study was obtained from the ethics research committee of the university (ERC_SU_20210057 dated 03-11-2021).

## Authors contributions

Conceptualization, KA, SC and AS; Data Collection, AS, BR and BA; Methodology, KA, SC, AS and BA; Writing-Original Draft Preparation, AS, KA and BA; Writing- Review & Editing, AS, SC, KA and BA; Supervision, AS and BR; Project Administration, AS and SC; Funding Acquisition, AS, KA, SC and BA. All authors have critically reviewed and approved the final draft and are responsible for the content and similarity index of the manuscript.
